# Supplementary material for: Stool-based Xpert MTB/RIF assay for the diagnosis of pulmonary tuberculosis in children at a teaching and referral hospital in Southwest Ethiopia
Source: PLoS One. 2022 May 5;17(5):e0267661. doi: 10.1371/journal.pone.0267661 (PMC9070927; doi:10.1371/journal.pone.0267661)
Supplement: S1 Table — LJ = Lowenstein-Jensen, MTB = Mycobacterium tuberculosis, GA = gastric aspirate. (DOCX) [file pone.0267661.s001.docx]

**S1 Table. The stool Xpert and GA Xpert MTB detection rate compared to LJ culture and/or GA Xpert positivity (microbiological confirmation).**

|  |  | **LJ culture and/or GA Xpert** | | |  |
| --- | --- | --- | --- | --- | --- |
|  |  | **Positive, n(%)** | **Negative, n(%)** | **Total, n(%)** | **p-value** |
| **Stool Xpert** | **Positive, n(%)** | 10(100) | 0 | 10(100) | 0.000 |
|  | **Negative, n(%)** | 0 | 142(100) | 142(100) |  |
|  | **Total** | 10(6.6) | 142(93.4) | 152(100) |  |
| **GA Xpert** | **Positive, n(%)** | 8(100) | 0 | 8(100) | 0.000 |
|  | **Negative, n(%)** | 2(1.4) | 142(98.6) | 144(100) |  |
|  | **Total** | 10(6.6) | 142(93.4) | 152(100) |  |

LJ=Lowenstein-Jensen, MTB=*Mycobacterium tuberculosis*, GA= gastric aspirate
