# Supplementary material for: Stool-based Xpert MTB/RIF assay for the diagnosis of pulmonary tuberculosis in children at a teaching and referral hospital in Southwest Ethiopia
Source: PLoS One. 2022 May 5;17(5):e0267661. doi: 10.1371/journal.pone.0267661 (PMC9070927; doi:10.1371/journal.pone.0267661)
Supplement: S2 Table — MTB = Mycobacterium tuberculosis, GA = gastric aspirate. (DOCX) [file pone.0267661.s002.docx]

**S2 Table. Stool Xpert, GA Xpert and GA culture MTB detection rate compared to composite reference standard (confirmed and unconfirmed TB).**

|  |  | **Composite reference standard (CRS)** | | | |  |
| --- | --- | --- | --- | --- | --- | --- |
|  |  | **Confirmed TB, n(%)** | **Unconfirmed TB, n(%)** | **Unlikely TB, n(%)** | **Total, n(%)** | **p-value** |
| **Stool Xpert** | **Positive, n(%)** | 10(100) | 0 | 0 | 10(100) | 0.000 |
|  | **Negative, n(%)** | 0 | 10(7.0) | 132(93.0) | 142(100) |  |
|  | **Total** | 10(6.6) | 10(6.6) | 132(86.8) | 152(100) |  |
| **GA Xpert** | **Positive, n(%)** | 8(100) | 0 | 0 | 8(100) | 0.000 |
|  | **Negative, n(%)** | 2(1.4) | 10(6.9) | 132(91.7) | 144(100) |  |
|  | **Total** | 10(6.6) | 10(6.6) | 132(86.8) | 152(100) |  |
| **GA culture** | **Positive, n(%)** | 9(100) | 0 | 0 | 9(100) | 0.000 |
|  | **Negative, n(%)** | 1(0.7) | 10(7.0) | 132(92.3) | 143(100) |  |
|  | **Total** | 10(6.6) | 10(6.6) | 132(86.8) | 152(100) |  |
