# Supplementary material for: Stool-based Xpert MTB/RIF assay for the diagnosis of pulmonary tuberculosis in children at a teaching and referral hospital in Southwest Ethiopia
Source: PLoS One. 2022 May 5;17(5):e0267661. doi: 10.1371/journal.pone.0267661 (PMC9070927; doi:10.1371/journal.pone.0267661)
Supplement: Attachment — Submitted filename: Response to Reviewers.docx [file pone.0267661.s004.docx]

**Response to Editor and Reviewers**

We appreciate the editor and reviewers for the constructive comments which we have used to improve the quality of the manuscript. We have re-written some portions of the manuscript accordingly. We have carefully addressed the comments line by line as follows.

**Editor’s comments to the Author:**

**Response to Editor’s comments:**

**Comment 1**: Please ensure that your manuscript meets PLOS ONE's style requirements, including those for file naming. The PLOS ONE style templates can be found at https://journals.plos.org/plosone/s/file?id=wjVg/PLOSOne_formatting_sample_ main_body.pdf and <https://journals.plos.org/plosone/s/file?id=ba62/PLOSOne_formatting_sample_title_authors_affiliations.pdf> .

**Response 1:** We have rechecked our manuscript for the PLOS ONE’s requirement and revised it accordingly.

**Comment 2:** Further clarification on source of funding.

**Response 2:** Thank you for your feedback on the source of funding. No specific funding was obtained for this research work and we have included the following statement in the revised version under the “Funding sources’’ subheading: **‘***The authors received no specific funding for this work.***’**

**Comment 3:** We note that you have provided funding information that is not currently declared in your Funding Statement. However, funding information should not appear in the Acknowledgments section or other areas of your manuscript. We will only publish funding information present in the Funding Statement section of the online submission form. Please remove any funding-related text

**Response 3:** No funding information was presented in the acknowledgment part. Regarding comments raised on source of funding, we have addressed them above (**Response 2**). I feel that we don’t need to update the funding statement. The funding statement can still read as “No”.

**Comment 4:** We note that you have indicated that data from this study are available upon request. PLOS only allows data to be available upon request if there are legal or ethical restrictions on sharing data publicly.

**Response 4**: We have uploaded our data set as a supporting information file (**S1 File**). We have addressed this in the revised manuscript.

**Comment 5**: Please note that in order to use the direct billing option the corresponding author must be affiliated with the chosen institute. Please either amend your manuscript to change the affiliation or corresponding author, or email us at plosone@plos.org with a request to remove this option.

**Response 5:** As it has been indicated in the manuscript cover page, the corresponding author is affiliated to Mycobacteriology Research Center, Jimma University, Jimma, Ethiopia.

**Comments 6:** PLOS requires an ORCID iD for the corresponding author in Editorial Manager on papers submitted after December 6th, 2016. Please ensure that you have an ORCID iD and that it is validated in Editorial Manager.

**Response 6:** Here is the ORCID iD of the corresponding author: <https://orcid.org/0000-0003-4751-2225> .

**Comment 7:** Your ethics statement should only appear in the Methods section of your manuscript. If your ethics statement is written in any section besides the Methods, please delete it from any other section.

**Response 7:** We have checked that ethics statement is mentioned only in the method section under the subheading “Ethical considerations”.

**Additional Editor Comments (if provided):**

**Response to Editor’s comment**

**Comment 1:** I agree with the reviewer's first two major comments and would also add that the authors should explicitly explain how they use this classification in their analysis under the "Data analysis" paragraph.

**Response 1:** We appreciate the editor and the reviewer for the interesting feedback on TB diagnostic classification**.** This is well accepted comment and we have adjusted the TB diagnostic classification as it was proposed by Graham *et al,* 2015 and described it in the methods section under the Diagnostic classification for analysis subheading*.*

**Comment 2**: Abstract/Methods, line 32 and all subsequent references to the composite reference standard (CRS): I would be better to say "clinical diagnosis with improvement after anti-TB treatment" instead of just "clinical improvement after anti-TB treatment."

**Response 2:** The editor’s feedback was found interesting and we have modified it accordingly.

**Comment 3:** Introduction, line 65: What do you mean by "most significant clinical sample?" The "most frequently used?" Please be more specific.

**Response 3:** We have changed the phrase to “the most frequently used’’.

**Comment 4:** Introduction, lines 66-70: The yield is not poor because of many young children's inability to expectorate, but it is poor because of the paucibacillary nature of most childhood TB. Children's inability to expectorate means that other methods to obtain specimens are needed.

**Response 4:** We partly agree with the Editor’s comment. No doubt that sputum is the most frequently utilized sample for the diagnosis of pulmonary TB. However, in most cases, children are unable to expectorate sputum specimen and when sputum is available, the yield is expected to be poor because of the paucibacillary nature of most childhood TB. This is included in the revised manuscript.

**Comment 5:** Introduction, line 71: The collection of sputum is not unsafe for children. These procedures are well tolerated; they are just resource-intensive, as the authors said, and also the capability to carry out these procedures may be lacking in many TB endemic areas. Please either justify or take out this reference to the procedures being unsafe.

**Response 5:** The feedback from the editor is found interesting and we have modified it accordingly.

**Comment 6:** Methods: Please add a sentence about the incidence of TB in Ethiopia, and please also briefly describe the setting of the study site (e.g. is it urban or rural? are most patients publicly insured? from rural settings? etc.)

**Response 6:** We have included these information accordingly in the revised version.

**Comment 7:** Methods, lines 94-96: Please specify that these participants were classified as the presumptive TB patients.

**Response 7:** We agreed with the Editor’s comment and we have specified accordingly.

**Comment 8:** Methods: Could the authors specify whether a minimum volume was collected for each respiratory sample?

**Response 8:** A minimum volume of 4ml for respiratory specimen and 3gm of stool specimen were collected. We have specified this in the revised manuscript.

**Comment 9:** Methods, lines 165-167: Do you mean "legal guardians" instead of "caregivers" (just wondering if only parents and legal guardians are allowed to sign informed consent)? Also, what was the minimum age for obtaining assent from the child?

**Response 9:** We have replaced the term “caregivers” by “legal guardians’’ in the revised manuscript. The minimum age for obtaining assent from children was 11 years age. We have indicated this in the revised manuscript.

**Comment 10:** Results, line 189: Please replace ". . . not detected in all . . ." with ". . . not detected in any…"

**Response 10:** We have replaced it accordingly.

**Comment 11:** Results and Discussion: In Methods, the authors state that one of the reference standards is positive culture; I assume this refers to positive culture from any respiratory specimen, not just gastric aspirate. I understand that none of the expectorated sputum samples were positive for *M. tuberculosis*. However, instead of referencing microbiological confirmation from GA as the reference standard, please reference microbiological confirmation from a respiratory specimen.

**Response 11:** The comment is well accepted and we have modified it accordingly.

**Comment 12:** In addition, I find it odd that none of the expectorated sputum samples were positive for *M. tuberculosis*. I would expect the older kids to be able to expectorate sputum, and they tend to have more adult-type TB. Could the authors provide an explanation? Were there a lot of epithelial cells indicating poor sample quality in the expectorated sputum samples?

**Response 12:** Thank you for the interesting and critical observation. As you rightly stated, older children could expectorate sputum and may produce more adult-type sputum. However, in the current study, none of the expectorated sputum samples was positive for MTB. This could be due to the presence of a lot of epithelial cells indicating poor sample quality in the expectorated sputum samples. However, we didn’t assess the quality of the sputum sample in the current study and we are unable to comment on it. This is addressed in the discussion section.

**Comment 13**: Figure 1 is good but could be improved with a few minor changes. I would take away the fist box "children visit JMC." I'd use the same term "presumptive TB cases" in the second box to be consistent w/ the rest of the manuscript. In the "results available" box I would take out the reference to the 10 that improved on ATT and instead add that to the culture
negative --> Xpert negative part (so you can indicate how many were classified as not TB and how many were classified as unconfirmed TB). I would also take out the box that says "152 patients were included in the analysis" because it's repetitive.

**Response 13:** We have modified the figure accordingly.

**Review Comments to the Author**

**Response to Reviewers’ comments:**

**Major**

**Comment 1:** The TB classification used was confirmed/probable/possible/not TB; the childhood TB classification was updated in 2015 (Graham *et al*.) adjusting their classification to this structure (Confirmed/Unconfirmed/Unlikely) would be important to allow comparison with other studies and any meta-analyses

**Response 1:** We are very grateful to the editor and the reviewer for the interesting feedback on TB diagnostic classification. As we have mentioned above, this is well accepted comment and we have adjusted the TB diagnostic classification as it was proposed by Graham *et al*, 2015 and described it in the method section under the Diagnostic classification for analysis subheading.

**Comment 2:** Similarly, the CRS is defined as a combining Unconfirmed TB with Confirmed TB in the definition of TB, and Unlikely TB as not TB. Again, would adjust definitions accordingly to be consistent with other studies. Would also move that up to the section on diagnostic classification

**Response 2:** This is also very well accepted comment from the reviewer. In the revised manuscript, this is corrected accordingly.

**Comment 3:**  Would note what measures of diagnostic accuracy were used in the methods, including sensitivity, specificity, positive and negative-predictive values.

**Response 3:** We agree with the reviewer’s comment and it is indicated as follow in the revised version of the manuscript. “*The Xpert sensitivity, specificity, positive, and negative predicted values and their 95% confidence interval (95%CI) were calculated compared to LJ culture and composite reference standard (CRS).*’’ We calculated the Xpert sensitivity, specificity, PPV and NPV using LJ culture and CRS as reference standard, though our main conclusion comes from using CRS.

**Comment 4:** In methods, please describe your approach to comparing were sputum and stool-based Xpert. Options include comparing 95% CIs, as well as McNamara’s paired test of proportions. Would be clear in the methods how similarities vs. difference in the two methods are defined, as that is one of your main conclusions.

**Response 4:** This is also a very fascinating feedback from the reviewer. Here we would like to clarify how the diagnostic difference or similarity is determined for the two methods (stool-Xpert vs GA-Xpert) in our study. We have revised our manuscript and the following statements were included “*Differences and similarities between the two methods (stool-Xpert versus GA-Xpert) is determined based on the 95%CI. Non-over lapping 95%CI dictated the presence of difference between the two methods and vice versa*.’’

**Comment 5:** Was smear microscopy performed on the respiratory specimens? If so, please indicate the results and stratify performance smear status

**Response 5**: Smear microscopy was not performed in our study. We expect that the yield from smear microscopy is minimal due to the paucibacillary nature of the disease and we didn’t perform it.

**Comment 6:** Was HIV testing done? Please indicate if done and HIV prevalence

**Response 6:** HIV test was not done. We tried to retrieve HIV test result from patients’ medical records but there were lots of missed results due to poor documentation practice.

**Comment 7:** In the 2 cases that died, how long were they taking the ATT? Would note the duration, because if soon after initiation, would exclude as unclassifiable

**Response 7:** The 2 cases that died were after 2 weeks of ATT initiation. We indicated the duration in the revised version of the manuscript. We thought that if it is TB, they would have shown some clinical improvements to the ATT within 2 weeks of treatment.

**Comment 8:** In determining the performance of stool Xpert for Confirmed cases, please use the 10 cases (9 culture confirmed, 1 GA Xpert positive) as opposed to 9 culture-positive only

**Response 8:** Many studies used culture alone as a reference standard when determining the diagnostic accuracy of new or already available diagnostic tools. Culture is one of the best method to detect TB and many scholars considered culture as the gold standard method. However, for pediatric and extra-pulmonary form of TB, culture alone may not be used as a reference standard as it misses a significant number of TB cases due to the paucibacillary nature of these disease. Our main interest here is to determine the performance of stool-Xpert using culture alone as a reference standard and to compare it with the diagnostic values when CRS is used as a reference standard. However, the main conclusion of our study was derived based on Stool-Xpert performance using CRS as a reference standard.

**Minor**
**Comment 1:**  To increase the work’s impact, would note earlier that a single step, centrifuge-free stool processing method was used earlier in the abstract/introduction, as this approach in particular is of interest in the field, including the KNCV kit.

**Response 1:** Well accepted complement from the reviewer. In the revised version, note was made on the single step, centrifuge-free stool processing method adopted from the KNCV TB Foundation.

**Comment 2:** There are some spacing issues, such as in line 61 “1.1million” and “in2018” in line 62. Please ensure spacing is okay throughout

**Response 2:** We accepted the reviewer’s comment. In the revised manuscript, this is corrected.

**Comment 3:** Were invalid stool results repeated?

**Response 3:** No; due to shortage of Xpert cartridges, we were unable to repeat invalid stool-Xpert results. We have indicated this in the manuscript.

**Comment 4:** Please indicate in the results that none of the children with clinically-diagnosed TB (probable/possible) were stool-Xpert positive.

**Response 4:** We agree with reviewer’s comment and in the revised manuscript, it was indicated as suggested by the reviewer.

**Comment 6:** PLOS authors have the option to publish the peer review history of their article (what does this mean?). If published, this will include your full peer review and any attached files.

**Response 6:** We agree to publish the peer review process.
