# Supplementary material for: Stool-based Xpert MTB/RIF assay for the diagnosis of pulmonary tuberculosis in children at a teaching and referral hospital in Southwest Ethiopia
Source: PLoS One. 2022 May 5;17(5):e0267661. doi: 10.1371/journal.pone.0267661 (PMC9070927; doi:10.1371/journal.pone.0267661)
Supplement: Attachment — Submitted filename: Response to Reviewers.docx [file pone.0267661.s005.docx]

**Response to Editor and Reviewers**

We appreciate the editor and reviewers for the constructive comments which we have used to improve the quality of the manuscript. As usual, we have re-written some portions of the manuscript accordingly. We have also carefully addressed the comments line by line as follows.

**Editor’s comments to the Author:**

**Response to Editor’s comments:**

**Journal Requirements:**

**Editor’s comment:** Please review your reference list to ensure that it is complete and correct. If you have cited papers that have been retracted, please include the rationale for doing so in the manuscript text, or remove these references and replace them with relevant current references. Any changes to the reference list should be mentioned in the rebuttal letter that accompanies your revised manuscript. If you need to cite a retracted article, indicate the article’s retracted status in the References list and also include a citation and full reference for the retraction notice.

**Response:** We have checked the references list and confirmed that it is correct and complete. None of the reference cited was retracted.

**Additional Editor Comments**

**Comment 1**: English editing service of the whole manuscript.

**Response 1:** We are very much grateful for the editor for English editing services. We found it very helpful to improve the quality of our manuscripts.

**Additional minor edits from the Editor**

**Comment 1:** To maintain consistency with the revised definition of the composite reference standard (CRS), which now includes “confirmed TB” and “unconfirmed TB” per the updated consensus case definitions by Graham, et al. and as recommended by the reviewer, please make the following changes:
**Comment 1a**: Abstract, lines 30-32: Diagnostic performance was calculated with reference to LJ culture and to a composite reference standard (CRS) of confirmed TB (by Xpert or culture) and unconfirmed TB (clinical diagnosis with improvement after anti-TB treatment).

**Response 1a:** We accepted the comment and corrected accordingly in the revised draft. **Shown in Line 31-32 of cleaned version**.

**Comment 1b:** Study procedures, lines 115:117: Study subjects were diagnosed with confirmed TB based on positive microbiological test results (positive LJ culture and/or positive Xpert on gastric aspirate or sputum), or with unconfirmed TB based on a clinical diagnosis and positive response to ATT.

**Response 1b:** We agree with the Editor’s suggestion and corrected accordingly. **Shown in Line 116-118 of cleaned version.**

**Comment 2:** Data analysis, lines 173-174: Please modify this sentence based on the reviewer’s comment to use culture and/or Xpert positivity as one of the reference standards.

**Response 2**: We found the editor’s comment important and corrected accordingly. **This is shown in the data analysis (Line 175) and result sections (Table 4 and S1 Table). Line 235-237.**

**Comment 3**: Results, lines 228-235 and relevant tables: Please modify based on the reviewer’s comment to use culture and/or Xpert positivity as one of the reference standards.

**Response 3:** The Editor’s/reviewer’s comment is clearly addressed in the revised version of the manuscript. **This is shown in the data analysis (Line 175) and result sections (Table 4 and S1 Table). Line 235-237.**

**Comments 4:** Background, line 60: Technically, SARS-CoV-2 has exceeded TB. I recommend saying “Tuberculosis (TB) is a global leading cause of death . . .

**Response 4:** It is modified as suggested by the Editor. **Shown in Line 61 of cleaned version.**

**Comment 5:** Discussion, lines 251-253: The lower sensitivity of stool Xpert compared to the CRS is because clinically diagnosed patients have paucibacillary disease. Would modify this sentence to be more precise. Also please use the abbreviation CRS to be consistent w/ the rest of the manuscript.

**Response 5:** We agree with the Editor’s comment and rewritten it accordingly. **Look at Line 263-264 of cleaned-revised version.**

**Comment 6:** There are minor grammatical errors throughout the manuscript. I have asked PLOS One if there is an editing service, but unfortunately, there is not. I have listed some corrections to word choice below (underlined), but I suggest the authors ask a native English speaker to review the paper before resubmission. I did not correct errors in punctuation.
**Comment 6a: Abstract**

i. Lines 21-22: . . . challenging mainly due to the difficulty of . . .
ii. Lines 29-30: . . . after application of a single-step, centrifuge-free stool processing method . . .
iii. Line 33: . . . 152 of whom had . . .
iv. Lines 35-36: . . . only ten had microbiologically confirmed (positive Xpert and/or culture) disease, and the remaining . . .
v. Lines 37-38: . . . compared to culture; however, the sensitivity . . .
vi. Lines 41-42: . . . sensitivity for stool is comparable to the sensitivity of Xpert for gastric aspirate . . .

**Comment 6b: Background**

i. Lines 72-73: . . . TB endemic settings. Thus, there is a need for . . .

**Comment 6c:** Study design and setting

i. Lines 94-96: . . . hospitals in the country. JUMC is a teaching and referral hospital that provides services for about 15 million people in its catchment area. Each year, JUMC reports approximately 15,000 inpatient hospital stays, 160,000 outpatient visits, and 11,000 emergency department visits.
ii. Lines 98-101: . . . an estimated annual incidence of 151,000 cases and an estimated 19,000 TB-related deaths, excluding HIV-associated TB deaths.

**Comment 6d:** Specimen collection and laboratory procedures

i. Line 129: . . . for research purposes . . .
ii. Lines 132, 134 and all subsequent instances: Please write out “gastric aspirate” instead of using the abbreviation “GA” as this abbreviation is not commonly used and unnecessary (will be easier for readers to understand the paper without going back to look for what “GA” means)
iii. Line 138: . . . followed by vigorous shaking and incubation for 15 minutes . . .
iv. Line 145 and all subsequent instances: Please do not abbreviate “min”; please write out “minutes”
v. Line 153: . . . from KNCV TB Foundation.

**Comment 6e:** Diagnostic classification for analysis

i. Lines 165-166: “CRS is defined as either confirmed TB or unconfirmed TB”; cases that met “unlikely TB” criteria were not classified as TB.

**Comment 6f:** Data analysis

i. Lines 175-176: “Non-overlapping 95% CIs indicated a difference between the two methods (stool Xpert vs. gastric aspirate Xpert).”

**Comment 6g:** Results

i. Line 189: . . . stool specimens.”
ii. Lines 189-191: Seventy-eight (51.3%) of 152 participants were females, and participants’ ages ranged from 7 months to
iii. 14 years (median 3 years). [Provide number here] (64.5%) of the participants were rural residents (Table 1).”
iv. Lines 192-197: Please report the number first, followed by the percentage in parentheses.
v. Line 195: “The majority of children . . .”
vi. Line 196: “Close to a third . . .”

**Comment 6h:** Diagnosis of TB

i. Lines 200-206 and throughout the manuscript: Please be consistent and use either “M. tuberculosis” or “MTB”
ii. Line 208: I don’t believe PTB was defined previously. Please write out “pulmonary TB” if it was not.
iii. Lines 209-210: We reviewed the medical records of the remaining 142/152 (93.4%) of participants who were microbiologically negative.

**Comment 6i**: Diagnostic performance of stool Xpert

i. Line 222: . . . a shortage of Xpert cartridges, we did not repeat . . .

**Comment 6j.** Discussion

ii. Line 239: The national guidelines for TB care . . .
iii. Line 241: respiratory specimens . . .
iv. Line 242: . . . for this population.
v. Line 249: microbiologically confirmed and/or clinically diagnosed TB.
vi. Line 283: . . . we did not assess the quality . . .

**Response 6 (6a-6j):** We are very grateful to the editor for his/her time and for correcting grammatical errors throughout the manuscript. The feedback from the editor is well accepted and considered in the revised version of the manuscript. We also shared our last version of the manuscript with language expertise and they made substantial English Language editions.

**Review #1 Comments to the Author**

**Response to Reviewers’ comments:**

**Comment 1:** I thank the authors for their thoughtful review of the comments, and have addressed the majority of them. For response 8, as the authors note culture alone will miss pediatric cases due to their paucibacillary disease. There are two goals of this analysis, one to provide an estimate of stool Xpert performance and the other to compare to respiratory specimen testing. In the first goal, the standard Confirmed TB definition should be used that includes Xpert results. For the second goal, it is reasonable to only use culture to allow comparison between the Xpert stool and GA to reduce bias. Would recommend presenting the results in these two ways; based on my understanding of the tables, this should still lead to 100% sensitivity and support their discussion line that all Xpert GA results were stool Xpert positive. Excluding Xpert from the main estimation results also confuses the comparison from microbiological to composite reference standard, as the CRS includes both culture and Xpert positive cases.

**Response 1:** We found that the feedback from the reviewer #1 is very interesting and the comment is addressed in method and results sections. In **S1 Table** (supporting information), stool Xpert and GA Xpert MTB detection rate compared to LJ culture and/or GA Xpert positivity (microbiological confirmation) was described. Moreover, the diagnostic performances of stool Xpert and GA Xpert using LJ culture and/or GA Xpert (microbiological confirmations) as one of the reference standards was indicated in **Table 4**. **This is shown in the data analysis (Line 175) and result sections (Table 4 and S1 Table). Line 235-237.**

**Comment 2:** 7. PLOS authors have the option to publish the peer review history of their article (what does this mean?). If published, this will include your full peer review and any attached files.

**Response 2:** We agree to publish the peer review process.

**Comment 3:** While revising your submission, please upload your figure files to the Preflight Analysis and Conversion Engine (PACE) digital diagnostic tool, https://pacev2.apexcovantage.com/. PACE helps ensure that figures meet PLOS requirements. To use PACE, you must first register as a user. Registration is free. Then, login and navigate to the UPLOAD tab, where you will find detailed instructions on how to use the tool. If you encounter any issues or have any questions when using PACE, please email PLOS at figures@plos.org. Please note that Supporting Information files do not need this step.

**Response 3:** We have checked our figure by PACE and found that it met PLOSONE requirements.
